# Analysis of RNA polymerase II ubiquitylation and proteasomal degradation

**DOI:** 10.1016/j.ymeth.2019.02.005

**Published:** 2019-04-15

**Authors:** Ana Tufegdzic Vidakovic, Michelle Harreman, A. Barbara Dirac-Svejstrup, Stefan Boeing, Anindya Roy, Vesela Encheva, Michelle Neumann, Marcus Wilson, Ambrosius P. Snijders, Jesper Q. Svejstrup

**Affiliations:** a Mechanisms of Transcription Laboratory, The Francis Crick Institute, 1 Midland Road, London NW1 1AT, UK; b Protein Analysis and Proteomics Laboratory, The Francis Crick Institute, 1 Midland Road, London NW1 1AT, UK

**Keywords:** RNA polymerase II, Degradation, Ubiquitylation, UV-irradiation

## Abstract

Transcribing RNA polymerase II (RNAPII) is decorated by a plethora of post-translational modifications that mark different stages of transcription. One important modification is RNAPII ubiquitylation, which occurs in response to numerous different stimuli that cause RNAPII stalling, such as DNA damaging agents, RNAPII inhibitors, or depletion of the nucleotide pool. Stalled RNAPII triggers a so-called “last resort pathway”, which involves RNAPII poly-ubiquitylation and proteasome-mediated degradation. Different approaches have been described to study RNAPII poly-ubiquitylation and degradation, each method with its own advantages and caveats. Here, we describe optimised strategies for detecting ubiquitylated RNAPII and studying its degradation, but these protocols are suitable for studying other ubiquitylated proteins as well.

## Highlights

• A variety of stimuli, including UV-irradiation, cause RNAPII ubiquitylation and degradation.
• Specific considerations are required for reproducible UV-irradiation of human and yeast cells.
• Dsk2/MultiDsk pulldown provides robust enrichment of ubiquitylated protein species.
• RNAPII degradation level depends on multiple variables.

## 1 Introduction

Transcription by RNA Polymerase II (RNAPII) is not a continuous process but rather involves frequent pausing, stalling or irreversible arrest, here collectively referred to as transcription stress. A number of elongation-stimulating factors have evolved to help RNAPII overcome these transcription-impeding events, yet resumption of transcription may not always be possible [1], [2]. Indeed, on its way along the gene, RNAPII faces obstacles, such as DNA damage, DNA-bound proteins, or DNA sequences that are intrinsically difficult to transcribe. Moreover, when occurring in the transcribed strand, bulky DNA lesions caused by genotoxic agents such as UV-irradiation, or chemicals such as cisplatin or 4-nitroquinoline 1-oxide (4-NQO) will completely block RNAPII progression [3], [4]. Transcription stress can also be induced independently of DNA damage, for example through depletion of the nucleotide pool by treatment with 6-azauracil (6-AU) in the yeast *Saccharomyces cerevisiae* [5], or through mutation of the gene encoding elongation factor TFIIS, which causes prolonged RNAPII stalling [6].

The RNAPII elongation complex is remarkably stable, which is crucial to ensure processivity of transcription under normal conditions. However, this great stability comes at a cost: just a single stalled RNAPII complex can block transcription of an entire gene. To avoid potentially detrimental consequences of persistently stalled/arrested RNAPII, cells have evolved several mechanisms to solve this problem. For example, damage-stalled RNAPII can act as a DNA lesion sensor, triggering transcription-coupled nucleotide excision repair (TC-NER) and removal of the lesion, which would allow resumption of transcription [7], [8]. However, RNAPII stalling/arrest, induced either by DNA damage or other means, has also been shown to induce extensive RNAPII ubiquitylation *in vivo*. Subsequently, poly-ubiquitylated RNAPII is subject to proteasomal degradation [6], [9], [10], [11], [12], [13].

Intriguingly, proteasome subunits have been found to associate with several yeast genes, particularly gene ends, in a transcription-dependent manner. Moreover, inhibition of proteasome proteolytic activity resulted in transcription readthrough beyond the transcription termination site [14]. More recently, RNAPII degradation has been proposed to play a role in hormone-induced enhancer decommissioning [15]. Additionally, some viruses have been shown to inhibit host cell transcription via inducing RNAPII degradation [16], [17], and RNAPII ubiquitylation can regulate splicing in yeast, in a manner independent of proteasomal degradation [18]. These observations suggest that RNAPII ubiquitylation and degradation may play roles beyond resolving arrested RNAPII.

In the context of transcription arrest, experiments in yeast suggested that RNAPII phosphorylated at serine 2 (S2) in the heptapeptide repeats of the C-terminal domain (CTD) is subject to poly-ubiquitylation and degradation, while phosphorylation of CTD S5 inhibited RNAPII poly-ubiquitylation *in vitro* [9]. This supports the idea that only elongating RNAPII is targeted for ubiquitylation and degradation when it encounters a transcription-blocking event and stalls. An important question that remains to be investigated is how the cells distinguish a stalled RNAPII from normal elongating RNAPII.

RNAPII is composed of 12 subunits; however, only RPB1, the largest and catalytic subunit, is subject to RNAPII arrest-induced degradation in yeast [19]. RPB1 ubiquitylation is a complex, multistep process, with multiple E3 ubiquitin ligases already having been implicated [2], [12], [20], [21], [22]. Briefly, Rsp5 (NEDD4 in human cells) is responsible for generating a K63 linked poly-ubiquitin chain on RPB1, which is then trimmed to mono-ubiquitylation by the Rsp5-associated deubiquitinating enzyme (DUB) Ubp2. In a reaction which is dependent on prior mono-ubiquitylation, the Elongin complex finally produces a K48-linked poly-ubiquitylation chain on RPB1 [12], [13], [20], [23], [24]. While this two-ligase system has been successfully reconstituted for yeast and mammalian factors *in vitro* [20], the human E3 ligase VHL has also been shown to ubiquitylate RPB1 *in vivo* [25]. Moreover, depletion of specific ligases *in vivo* results only in partial loss of ubiquitylation, or in lack of RPB1 ubiquitylation only at specific stages of the response – for example, depletion of NEDD4 abrogates RPB1 poly-ubiquitylation but only at early timepoints after UV-irradiation in human cells [12]. Together, these results suggest that two or more redundant pathways leading to RNAPII poly-ubiquitylation and degradation may be active, at least in human cells. Additionally, the yeast Def1 protein, which is not an E3 ligase itself, facilitates Rpb1 degradation upon DNA damage by enhancing recruitment of the Elongin complex to RNAPII [26]. Finally, Cdc48 (human p97), in complex with Ubx4/5, recognizes and extracts poly-ubiquitylated Rpb1 from chromatin, in order for it to be degraded by the proteasome [27], [28]. To add to this complexity, the DUB Ubp3 has been shown to digest poly-ubiquitin chains on yeast Rpb1 [29], presumably to proofread and rescue Rpb1 from degradation. Taken together, it is clear that although great progress has been made in outlining the pathways and mechanisms responsible for RPB1 ubiquitylation and degradation, it is possible that not all the key players in this complex process have been identified. It thus remains to be clarified or investigated which E3 ligases, DUBs and auxiliary factors are involved in human RPB1 ubiquitylation/deubiquitylation, and how their complex interplay is achieved.

In order to help address these questions, robust and reliable methods to study ubiquitylation are required. Here, we discuss some of these techniques and provide detailed protocols for tracking human and yeast RPB1 ubiquitylation and degradation. We also briefly address future perspectives, such as mapping individual ubiquitylation sites and generating suitable cell model systems. Importantly, although the approaches described were designed specifically to investigate RNAPII ubiquitylation, they can easily be modified to investigate ubiquitylation of other proteins as well.

## 2 Inducing RNAP II ubiquitylation and degradation – reagents and equipment

As mentioned above, many different kinds of stimuli can cause ubiquitylation and degradation of RNAPII largest subunit, RPB1. Among those cues, UV-irradiation is probably the best studied. Below, we outline the equipment, protocols and considerations needed for UV-irradiating human and yeast cells. We also provide a brief overview of other protocols to trigger RPB1 ubiquitylation and degradation.

### 2.1 Equipment for UV-irradiation

Any device capable of emitting a regulated dose of UV light can potentially be used to irradiate mammalian or yeast cells grown in culture. UV light of any wavelength (UVA = 315–400 nm, UVB = 280–315 nm, UVC = 100–280 nm) can thus induce the formation of DNA lesions (cyclobutane pyrimidine dimers (CPDs) and (6-4)-photoproducts (6–4)PPs) [30]. When found in the transcribed strand of active genes, CPDs and (4-6) PPs will cause RNAPII stalling [3], [4]. Here, we focus on UVC, as it is the most frequently used UV-irradiation type in the literature.

**Fig. 1:**
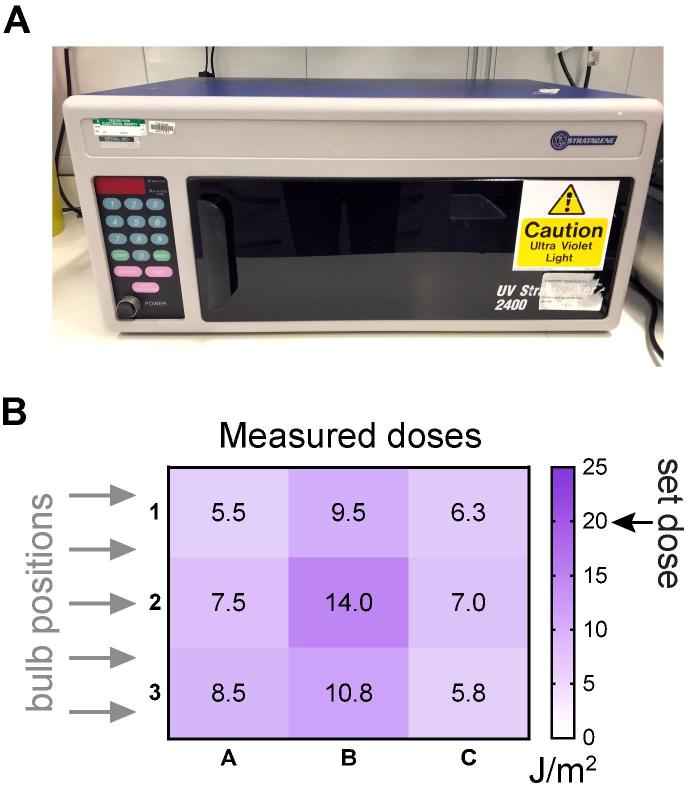
Equipment for UV irradiation of cells. (A) A typical UV crosslinker (Stratalinker) device. (B) Variability of exposure across a typical UV crosslinker (Stratalinker) surface. Desired doses were set to 20 J/m^2^ UVC and actual emitted doses were measured with a UV meter at 9 different areas of the crosslinker surface. The average of four measurements is indicated. The positions of the device’s light bulbs are indicated by arrows.

The most commonly available irradiation devices are those routinely used for crosslinking nucleic acids to membranes, for example UVC crosslinkers (Fig. 1A). Although these crosslinking devices can often be sufficient, there are important considerations to bear in mind. First, these devices were designed primarily with the purpose of providing a very high dose of UVC-irradiation, for the crosslinking of molecules to membranes. As such, their reliability in the low UV-dose range required for irradiation of live cells may not be optimal. Particularly, we noticed that UV lamps/crosslinkers suffer from relatively high levels of inconsistency when providing the low doses of UVC (5–20 J/m^2^) typically used to irradiate human cells, and that they give disconcertingly non-uniform irradiation across the exposed surface (Fig. 1B). As an alternative to commercial UV crosslinkers, custom-made UV-irradiation devices can be built, such as UV boxes with a longer, adjustable distance to the target material. Such boxes are particularly useful for irradiating yeast cells, as they allow easy agitation/mixing of the sample during irradiation so that uniform exposure of yeast cells to the UV source is obtained.

A key component for all experimental settings is a UV meter (e.g. VLX-3W). Whether using a UV-crosslinker or a UV box, the UV meter probe can be inserted in the irradiation chamber to measure the actual UV dose given. Due to the spatial variability in the actual given doses by many UV sources, the experimental setup needs to be carefully tested by measuring the UV dose across the surface of the irradiation device to determine not only the uniformity of irradiation but also the maximum size of the surface that can be accurately and uniformly irradiated (e.g. see Fig. 1B).

Different UV-irradiation devices will achieve a given UV dose in different amounts of time – for example, total emission of 20 J/m^2^ energy may take 2–3 s in a UV crosslinker, and ∼10 s in a UV box with greater distance between the UV lamp and the sample. In the context of RPB1 ubiquitylation and degradation, we are not aware of any differences in results whether cells receive a similar total dose over a shorter or longer period of time.

### 2.2 Notes before starting

• Warm up the UV-irradiation device before use (10 min for the UV box, or give two pulses of 2500 J/m^2^ with crosslinkers).
• Make sure the expected doses are correct by using a UV-meter.
• The original media will need to be added back to the cells, therefore it is important to carefully remove and store it for reuse.

### 2.3 UV-irradiation of human cells

At the time of irradiation, the cells should be around 60–80% confluent and equally spread across the dish surface. Prior to irradiation it is necessary to remove the culture medium entirely, and also to take the lid of the dish off for irradiation (UVC rays do not pass through the plastic). The experimenter should aim to work fast and avoid leaving the cells without medium for more than a few minutes. The dishes should be transferred from the tissue culture hood to the UV-irradiation device with the dish lid kept on, to maintain sterility. The lid should be taken off just before irradiation, inside the UV crosslinker chamber. The lid should be kept facing down on a clean, sterilised surface. If the samples will be used for assaying transcription, we recommend replenishing each sample of cells with the medium they were grown in before irradiation. Adding fresh medium can cause a “boost” in transcription and potentially skew the results [31].

### 2.4 UV-irradiating yeast

Yeast cells should be in early log phase (5 × 10^6^–2 × 10^7^ cells/ml) at the time of UV-irradiation. 25 μg/ml cycloheximide may be added 5 min before starting the experiment (see below). The cells should be washed and resuspended in PBS or saline for UV-irradiation. The original media should be saved as it will be re-added to the yeast cells after treatment. We typically start with a 200 ml culture per condition. The culture is split in two, with one half irradiated and the other half saved as the negative control. The cells are concentrated 5 times for the irradiation such that 100 ml of culture is resuspended in 20 ml of PBS/Saline, and irradiated in a pyrex dish with mixing of the sample during irradiation, if possible. The UVC dose we give is usually 300 J/m^2^ (over approximately 30 s), as measured with the UV meter. Immediately after the samples have been irradiated they are collected by centrifugation and resuspended in their original media for recovery. At this point, it is important to keep the recovery cultures in a shaking incubator in the absence of light, to ensure that activation of photolyase is prevented [32]. Note: if possible, use YPD instead of minimal media to grow the cells, as this tends to give more reproducible and robust results.

### 2.5 UV meter: the importance of monitoring the dose

Most UV-induced phenomena are dose dependent (see below). Considering that some UV devices are inconsistent in providing a given dose, it is of utmost importance to independently monitor the emitted dose of irradiation with a UV radiation meter in every experiment. While there are many UV radiation meters on the market, it is important for it to fulfil the following criteria:

• The meter should be able to measure the wavelength of the given UV-irradiation device.
• The meter should be able to measure cumulative UV exposure (over a period of time).
• The probe of the meter should have a cable of appropriate length to be able to fit into the corresponding irradiation device’s chamber.

We have successfully used UV radiation meter VLX-3W with probe SX254, with UVC crosslinkers and a UVC box.

### 2.6 Troubleshooting

Measuring low actual doses, or very variable actual doses between samples. Potential causes:

• Device not warmed up properly. Warm up the device as recommended in “Notes before starting”, or try warming up for a longer period of time.
• Old bulbs. Replace bulbs.
• If neither of the above helps, the device may not be suitable for irradiation of live cells.
• For multi-well plates (24-well to 384-well) alternative irradiation devices may need to be built to achieve consistent exposure across samples. We have successfully constructed and used UV conveyor belts that operate by having a single UVC light source in the centre of the device’s chamber, and a moving conveyor belt that will carry the sample through the chamber with constant speed [33].

### 2.7 Other stimuli that induce RPB1 degradation

Apart from UV-irradiation, other agents are also known to induce RPB1 ubiquitylation and degradation. The common feature is that they induce transcription stress either by creating transcription-blocking DNA damage (e.g. UV, cisplatin, 4-NQO), by blocking RNAPII activity (e.g. alpha-amanitin) or by depleting the NTP pool (e.g. 6-AU). The commonly used dose ranges for these agents are listed in Table 1.

**Table 1:** Agents that induce RPB1 poly-ubiquitylation and degradation, and the range of doses used in the literature (recommended dose in brackets).

Reagent	Dose (Human)	Dose (yeast)
UVC-irradiation	2–100 J/m^2^ (typically 5–20 J/m^2^)	Up to 300 J/m^2^
Cisplatin	5–50 μM for 1 h	Not used
4-NQO	0.2–5 μM for 1 h	50 μM, 30 min
6-AU	Not tested	2 mM, 2–3 h
Alpha amanitin	2–50 μM (10 μM) for 4 h	Not used

**Fig. 2:**
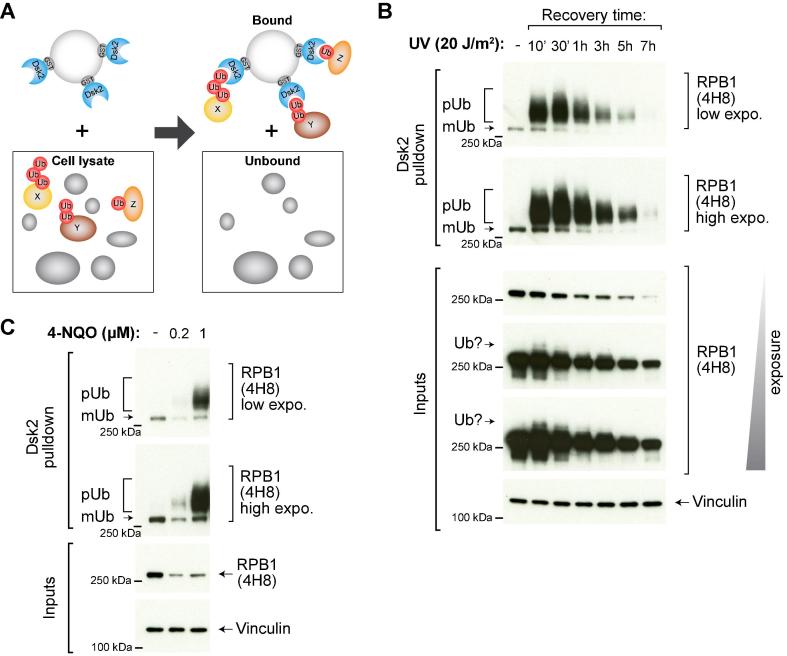
Enrichment of ubiquitylated RNAPII species. (A) A sketch illustrating the principle of Dsk2 pulldown strategy (the same principles apply to MultiDsk). Dsk2 binds the ubiquitin moiety on proteins and this property is exploited to enrich ubiquitylated proteins from the extract. Note that, if used as indicated in text, this method is capable of depleting the extract of ubiquitylated proteins. (B) Kinetics of RNAPII ubiquitylation upon UV irradiation in human cells. HEK293 TRex flpIn cells were irradiated with 20 J/m^2^ UVC, and samples were taken at different timepoints after irradiation. Dsk2 analysis was performed as described in text. Note that different exposures of the same Western blot membranes are shown. Approximate positions of the protein markers are indicated on the left. mUb, mono-ubiquitylated RPB1. pUb, poly-ubiquitylated RPB1. “Ub?”, slower migrating RPB1 band, which may represent ubiquitylated species in the extract. (C) Analysis of RNAPII ubiquitylation upon 4-NQO treatment in HEK293 TRex flpIn cells. The cells were treated with the indicated doses of 4-NQO for 1 h, and Dsk2 analysis was performed as described in the text.

For practical reasons, it may be easier to use one of these chemicals rather than UV-irradiation to induce RPB1 ubiquitylation and degradation. For example, 4-NQO is typically used in yeast when performing time-course experiments, as UV-irradiation of yeast requires a significant amount of time to carry out, and might not be possible to perform for multiple different strains or conditions in a time-course set-up. We tested 4-NQO treatment in human cells and found that similarly to yeast, it induces RPB1 poly-ubiquitylation and degradation, but at much lower doses than those typically used for yeast (Fig. 2C). While all of these agents will induce RPB1 ubiquitylation and degradation, it is unclear if they do so via the exact same molecular mechanism (e.g. using the same ubiquitin ligase pathways or the same ubiquitylation sites on RPB1), although, to date, we have not observed any inherent differences between them.

## 3 Detection of ubiquitylated RNAPII

### 3.1 Background

Mono-, oligo- or poly-ubiquitylation of proteins can often be detected by simple Western blot analysis as additional, slower-migrating bands and smears, for example for the well-known DNA damage-induced mono-ubiquitylation of histone H2AX [34], [35]. However, for RPB1 this is not the case because *i)* RPB1 is a very large protein so it is difficult to resolve and visualize these “extra” bands, *ii)* only a small fraction of RPB1 molecules are ubiquitylated at any given time, even after high doses of UVC-irradiation.

In both mammalian and yeast cells, a faint band can sometimes be seen above the phosphorylated RPB1 band in UV-irradiated samples (for example Fig. 2B, overexposed Input lanes, 4H8), but it would be challenging and likely unreliable to quantify this presumed ubiquitylation smear and compare between conditions. For these reasons, prior enrichment of ubiquitylated RPB1 molecules is required. This can be achieved by pulldown of all ubiquitylated proteins from the lysate and then probing for RPB1 using specific antibodies in Western blot analysis [12].

We note that other methods have been utilized where RPB1 is immunoprecipitated and then blotted with anti-ubiquitin antibodies. However, these protocols may potentially detect not only ubiquitylated RPB1 but also any other ubiquitylated proteins that co-immunoprecipitate with RPB1. Furthermore, other alternative procedures sometimes enrich (hexahistidine- or epitope-) tagged ubiquitin and then probe for RPB1. This approach is reliable, but sometimes impractical as it requires the generation of cell lines which express tagged ubiquitin. Below we describe the principles of the methods we regularly use to easily and reliably detect ubiquitylation of RNAPII. Importantly, these approaches can actually be used to detect any ubiquitylated protein for which a specific antibody is available.

### 3.2 Method: enrichment of ubiquitylated proteins using Dsk2 or MultiDsk pulldown

The budding yeast Dsk2 protein contains an UBA domain, which binds ubiquitin with relatively high affinity [36]. This has been exploited to generate GST-Dsk2 affinity resins that can enrich native, ubiquitylated proteins from cell extracts (Fig. 2A) [12]. A further development of this principle was TUBES [37], or MultiDsk resin [38], which contain an array of four (TUBES), or five (MultiDsk) UBA domains, respectively. We have worked extensively with MultiDsk and it displays a dramatically higher affinity for ubiquitin and ubiquitin chains than one Dsk2 domain alone, to the extent that ubiquitylated proteins can effectively be depleted from the extract [38]. While Dsk2 protein appears to have some preference towards binding K48-linked ubiquitin chains [36], [39] and is therefore highly suitable for detecting poly-ubiquitylated proteins destined for proteasomal degradation, MultiDsk does not significantly discriminate between different types of ubiquitin chains, and can also effectively detect mono-ubiquitylated proteins [38]. We have used both Dsk2 and MultiDsk to successfully enrich ubiquitylated RPB1 species [12], [38].

Generation of GST-MultiDsk resin has already been described in detail [38], and is slightly more challenging than generating GST-Dsk2 resin due to the relatively insoluble nature of the MultiDsk protein [38]. Below we provide a detailed protocol for the generation and use of GST-Dsk2 affinity resin for enrichment of ubiquitylated RPB1, and also highlight the differences between this protocol and that used for generation of MultiDsk resin. MultiDsk is also available from several commercial providers.

### 3.3 Preparation of GST-Dsk2 affinity resin

#### 3.3.1 Reagents and equipment needed

• pGEX3-Dsk2 plasmid [12].
• One Shot BL21(DE3) or One Shot BL21(DE3) Star competent bacterial cells, and all the necessary equipment for bacterial transformation, growth and induction.
• PBSA buffer, protease inhibitors, Triton X-100, DTT.
• Glutathione sepharose beads, 50% packed bead volume solution.
• Sodium azide solution.
• 50 ml falcon tubes, 200 ml glass beakers.
• Tip probe sonicator (e.g. Branson Digital Sonifier 250).
• Centrifuge.
• For specific details of the reagents and equipment please refer to Appendix.

#### 3.3.2 Bacterial transformation, growth and lysate preparation:

• *Day 1*: Transform One Shot BL21(DE3) or One Shot BL21(DE3) Star competent bacterial cells with pGEX3-Dsk2 plasmid according to the manufacturer’s instructions, and plate cells on ampicillin selection plates. Keep at 37 °C overnight.
• *Day 2*: pick a single colony and inoculate into 20 ml of LB containing 100 μg/ml ampicillin (LB_amp_) and shake at 37 °C at 200 rpm overnight. This is the pre-inoculum culture.
• *Day 3:*
  o Inoculate 300 ml of LB_amp_ with 5 ml of the pre-inoculum in 2L Erlenmeyer flask. Shake at 37 °C at 200 rpm.
  o Regularly take 1 ml aliquots to track bacterial growth by measuring absorbance at 600 nm (OD_600_).
  o When the OD_600_ reaches 0.8, take a 1 ml aliquot as the “non-induced” sample (centrifuge and keep on ice), and then induce the culture with 1 mM (final concentration) of IPTG.
  o Shake at 30 °C at 200 rpm for 4 h.
  o Take a 1 ml aliquot as the “induced” sample.
  o Aliquot the culture into 50 ml falcon tubes (or other appropriate vessels) and centrifuge at 4,500 rpm for 10 min to pellet bacteria. Also pellet the “non-induced” and “induced” samples by centrifugation.
  o Remove the supernatant and snap-freeze the pellets in liquid nitrogen. Store at −80 °C.
• *Day 4*:
  o Defrost the pellets from the 300 ml culture (usually 6 × 50 ml falcon tubes) quickly at room temperature then transfer to ice.
  o To each falcon tube (pellet derived from 50 ml culture), add 15 ml of cold PBSA containing protease inhibitors and resuspend the pellet completely by careful pipetting, or vortexing. Avoid denaturing proteins, often signified by bubbles in the mixture. Combine all 6 samples (90 ml total) into one 200 ml glass beaker.
  o Sonicate with a tip probe sonicator (Branson Digital Sonifier 250) at 33% output, with 15 s ON, 30 s OFF pulses, for a total ON pulse duration of 10 min. Keep the sample on ice at all times. If a different sonicator is used, the conditions for cell lysis will have to be optimised.
  o Add Triton-X100 to a final concentration of 0.5%, mix gently.
  o Incubate on ice for 30 min.
  o Transfer the sonicated lysates to appropriate vessels and centrifuge at 12,000 rpm for 10 min at 4 °C to remove debris. Alternatively, 4,500 rpm for 30 min in falcon tubes can be used.
  o Transfer the supernatant to new 50 ml falcon tubes on ice (distribute 90 ml of cleared lysate as 3 × 30 ml aliquots). Also take a 1 ml aliquot as the “lysate” sample. Successful expression of the GST-Dsk2 protein may be tested by gel electrophoresis and staining with Coomassie Blue before proceeding.
  o Resuspend the glutathione sepharose beads well and take 3 ml of suspension into a fresh tube (this corresponds to 1.5 ml packed bead volume). Spin at 500 g for 5 min at 4 °C and remove supernatant carefully. Wash once with cold PBSA, and then resuspend in 3.3 ml cold PBSA.
  o For binding of GST-Dsk2 to the beads, add 1 ml of well-resuspended glutathione sepharose bead solution from the previous step to each 30 ml of cleared lysate. Also add DTT to a final concentration of 2 mM. Rotate gently in the cold room for at least 4 h or overnight.
• *Day 5*:
  o Spin binding reactions at 500 g for 5 min at 4 °C. Remove and save the supernatant as the unbound fraction.
  o Wash the beads twice with ice-cold PBSA, 0.1% Triton X-100, containing protease inhibitors. During one of the washes, combine the pellets from the 3 falcon tubes into one.
  o Wash once more with PBSA *without* Triton X-100, but containing protease inhibitors.
  o Add 30 ml of PBSA containing protease inhibitors and 0.02% sodium azide to the prepared Dsk2 beads and store at 4 °C. In our hands, the beads can be stored for long periods of time (months) without losing efficiency.
• *Day 6*:
  o To check the efficiency of the Dsk2 bead preparation, pipet 100 μl of “non-induced”, 100 μl of “induced” and 100 μl of “lysate” into Eppendorf tubes. Also pipet 250 μl of prepared Dsk2 bead suspension (12.5 μl packed beads) into a tube, spin at 500 g for 5 min at 4 °C, and remove the supernatant. Add a corresponding amount of Laemmli buffer containing DTT or β-mercaptoethanol to each sample including the pelleted Dsk2 beads, and boil at 96–98 °C for 5 min. Spin at max speed for 5 min to remove debris or beads. Transfer supernatants to new tubes.
  o Run the samples on a polyacrylamide gel (typically 4–15%) and stain the gel with Coomassie or similar. GST-Dsk2 protein should run at ∼65 kDa, and some free GST band may also sometimes be seen at ∼28 kDa.

### 3.4 Preparation of MultiDsk affinity resin

MultiDsk plasmid (pGST-MD) is propagated and MultiDsk protein expressed as described for GST-Dsk2 in Section 3.3 up to day 4, with the exception that IPTG is added when the OD_600_ reaches 0.6. Since the MultiDsk protein is found in inclusion bodies, we use a different method of lysis and purification as described below.

• *Day 4*:
  o Defrost the pellet from the 300 ml culture quickly at room temperature then transfer to ice. Add 20 pellet volumes of STE buffer (10 mM Tris pH 8.0, 100 mM NaCl, 1 mM EDTA, protease inhibitors) containing lysozyme at 0.1 mg/ml and resuspend the pellet completely by careful pipetting, or vortexing. After 15 min on ice, add sarcosyl (N-lauryl sarcosine) to a final concentration of 1.5% in order to denature the protein.
  o Sonicate with a tip probe sonicator (Branson Digital Sonifier 250) at 20% output, with 15 s ON, 30 s OFF pulses, for 4 cycles. Keep the sample on ice at all times. If a different sonicator is used, the conditions for cell lysis will have to be optimised.
  o Centrifuge at 10,000 g for 5 min to remove insoluble material. Save the supernatant and take an aliquot for analysis.
  o Add Triton X-100 to the supernatant at a final concentration of 3%. Triton X-100 masks the sarcosyl by forming mixed micelles allowing the protein to refold.
  o Add this lysate to glutathione beads, pre-equilibrated in STE buffer. Use approximately 1 ml resin per 1 ml bacterial pellet starting material. Add DTT to 2 mM. Rotate gently in the cold room for at least 4 h or overnight.
• *Day 5*:
  o Spin bead-binding reactions at 500 g for 5 min at 4 °C. Remove and save the supernatant as the unbound fraction.
  o Wash the beads twice with ice-cold STE containing 500 mM NaCl and 0.1% Triton X-100, followed by a wash in the same buffer at 50 mM NaCl.
  o Wash twice with PBSA. Then add 30 ml PBSA.
  o The beads can be stored in PBSA, 0.02% sodium azide at 4 °C. In our hands, the beads can be stored for long periods of time (months) without losing efficiency.
• *Day 6*:
  o Check the efficiency of MultiDsk bead preparation, as for Dsk2 beads. GST-MultiDsk protein should run at ∼60 kDa, and some free GST band may also sometimes be seen at ∼28 kDa.

### 3.5 Generating cell lysates from human and yeast cells

#### 3.5.1 Human whole cell lysates

• Seed at least one 10 cm dish per condition. Apply treatments as required for your model system (e.g. siRNA, UV-irradiation, drug treatment, etc). At the time of collection, the cells should ideally be 60–80% confluent.
• *Note*: it is not necessary to use proteasomal inhibitors to observe RPB1 poly-ubiquitylation in human cells, if the optimal experimental timepoints are chosen (see Section 3.8).
• Aspirate the media and add the appropriate amount of PBS (e.g. 1–2 ml for a 10 cm or 15 cm dish).
• Immediately scrape cells off and transfer to a tube.
• Spin at 1300 rpm for 3 min, remove supernatant. The pellets can be flash-frozen in liquid nitrogen at this point and stored at −80 °C.
• Add the appropriate volume of TENT buffer (50 mM Tris-HCl pH 7.4, 2 mM EDTA, 150 mM NaCl, 1% Triton X-100) containing protease inhibitors, phosphatase inhibitors and 2 mM of N-ethylmaleimide [NEM; a potent and inexpensive cysteine protease (e.g. DUB) inhibitor] to the pellet. We typically use 1 ml of TENT buffer for a pellet from a 10–15 cm dish. Note that the NEM stock solution (200 mM in ethanol) should be made up fresh every time.
• Leave on ice for 10 min.
• Sonicate in a 4 °C water bath sonicator (Bioruptor) at high power, with 30 s ON and 30 s OFF pulses, for a total duration of 7 min, or by using a single sonication pulse (10 s at 20% output) with the tip-probe sonicator (Branson). Alternative sonicators can be used but the conditions would need to be optimised.
• Transfer to Eppendorf tubes and centrifuge at maximum speed (14,000 rpm) for 5 min to remove debris.
• Transfer the supernatant (lysate) to a new tube. Quantify the protein concentration using a Bradford assay or similar.

#### 3.5.2 Yeast whole cell lysates

• Harvest approximately 3 × 10^8^ yeast cells by centrifugation. At the time of collection, the cells should ideally be in early log phase (5 × 10^6^–2 × 10^7^ cells/ml).
• *Note:* MG132 is not added to yeast cell media as yeast cells are impermeable to it, but it is used later in the lysis buffer to prevent protein degradation in the lysates (see below).
• Wash once in PBS before flash-freezing the pellets in liquid nitrogen.
• Thaw pellets and resuspend in 800 μl buffer (150 mM Tris-acetate pH 7.4,100 mM potassium acetate, 1 mM EDTA, 0.1% Triton X-100, 10% glycerol, 2 mM NEM, 10 μM MG132 and protease inhibitors).
• Add roughly 500 μl 0.5 mm diameter glass beads (BioSpec Products) and disrupt the cells using a FastPrep-24 cell homogenizer (MP Biosystems), using 6 rounds of beating at an intensity of 5.5, for 30 s each. Incubate samples on ice for 1 min between disruptions, to reduce heating of the sample with an additional 5-minute ice incubation after 3 rounds. If a different method of homogenization is used, the conditions for cell lysis will have to be optimised.
• Clarify the extracts at 20,000 g for 10 min. Save supernatant and repeat centrifugation until the lysate is clear.
• Determine the protein concentrations of the extracts using the Bio-Rad protein assay or similar.

### 3.6 Enrichment of ubiquitylated proteins using Dsk2 beads

#### 3.6.1 Dsk2 pulldown with human cell lysates:

• *Day 1*:
  o We typically use 0.5 ml of GST-Dsk2 (or MultiDsk) bead suspension (equivalent to 25 μl packed beads) prepared as described in Section 3.3, to deplete/enrich ubiquitylated proteins from 1 mg of whole cell protein extract.
  o Keep both beads and protein samples on ice at all times.
  o For each cell lysate sample, pipet 1 mg of total protein into 2 ml safe-lock Eppendorf tube and slowly adjust all samples to the same volume with TENT buffer containing protease inhibitors, phosphatase inhibitors and 2 mM freshly made NEM. Typically, the final sample volume should be between 700 μl and 1 ml.
  o *Note:* it is possible to use less than 1 mg of protein per pulldown – we have tested amounts down to 300 μg. In this case, the volume of Dsk2 beads and the total reaction volume should be scaled accordingly.
  o Prewash the beads in bulk. Spin beads at 500 g for 5 min at 4 °C, remove supernatant and wash once with TENT buffer containing protease inhibitors, phosphatase inhibitors and 2 mM NEM (see Section 3.5.1).
  o *Note:* sepharose beads are very easily disturbed when removing the supernatant. It is therefore better to leave some supernatant behind than to accidentally remove some of the beads from the pellet.
  o Gently resuspend beads in a smaller volume of TENT buffer containing protease inhibitors, phosphatase inhibitors and 2 mM NEM (typically 220 μl per sample). Avoid making bubbles.
  o Aliquot the same volume (typically 200 μl) of well-resuspended Dsk2 bead slurry to each sample.
  o Rotate on a turning wheel/rotator (low to moderate speed) in the cold room for several hours to overnight.
• *Day 2*:
  o Spin the samples at 500 g for 5 min at 4 °C, remove supernatant and save as “unbound” fraction. Wash the beads carefully twice with 1 ml of TENT buffer containing protease inhibitors, phosphatase inhibitors and 2 mM NEM.
  o Wash the beads carefully once with 1 ml of PBS containing protease inhibitors, phosphatase inhibitors and 2 mM NEM. Spin at 500 g for 5 min at 4 °C and remove as much supernatant as possible. Re-spin a few times if necessary. At this point, any remaining liquid may also be removed with a fine pipet-tip.
  o To each bead sample, add 40 μl of Laemmli buffer containing DTT or β-mercaptoethanol, mix by brief vortexing, and boil at 96–98 °C for 5 min.
  o Spin the samples and save supernatant which now contains the enriched, ubiquitylated proteins (“bound” fraction). Samples can be run on SDS gels at this point or can be frozen.
  o *Note:* it is possible to elute bound proteins from the Dsk2/MultiDsk beads by the addition of 50 mM Phosphate buffer pH 8.1, 300 mM KCl, 10% glycerol, 1xPIs, 0.05% Triton X-100, 15 mM Glutathione, pH adjusted to >8.1 [38]. This is only required if the bound fraction is to be used for analysis other than WB (e.g. mass spectrometry or further binding assays).
  o *Note:* “unbound” and “bound” fractions can be run on the same gel, and a Western blot with anti-ubiquitin antibodies can be used to determine the efficiency of the pulldown (e.g. see [38]). In our hands, with the conditions outlined here (and overnight incubation for binding to beads), an apparently near-complete depletion of poly-ubiquitylated proteins from human extracts can be achieved. Using more than 0.5 ml of Dsk2 bead suspension (25 μl packed beads) per reaction does not yield markedly better enrichment of ubiquitylated proteins. However, the experimenter is advised to check the efficiency of their own Dsk2 bead preparation to determine optimal bead-to-sample ratios.

#### 3.6.2 Dsk2 pulldown with yeast cell lysates:

Dsk2 pulldowns from yeast extracts are carried out as with mammalian cell extracts, except for the following points:

• Typically, Dsk2 pulldowns with yeast extracts are performed with 2 mg of extract in a total volume of 1 ml and 30 µl of packed affinity resin. Adjust volumes with lysis buffer containing protease inhibitors, NEM and MG132.
• The extract and resin are incubated at 4 °C for *two hours* only; overnight incubations result in degradation of the Dsk2 protein.
• The resin should be washed once in the extract buffer, followed by two washes in the extract buffer with 300 mM potassium acetate, before a final wash in extract buffer.
• Elution should be performed as for mammalian cells.

### 3.7 Western blot analysis of Dsk2- and MultiDsk- enriched fractions

• The input and Dsk2/MultiDsk-enriched samples should be gel-analysed with enough spacing between them – we recommend running the input and Dsk2-enriched samples on different sides of the gel as the signal will often be much stronger in the inputs. We routinely use ∼5 μg of human cell lysate as input and 10–20 μl of the “Dsk2-bound” fraction (see previous section).
• To resolve Dsk2-enriched, ubiquitylated RPB1, we typically use a 3–8% Tris-Acetate gel, and continue electrophoresis until the 75–100 kDa marker runs out. Alternatively, 4–15% TGX gels can be used, again until the 75–100 kDa marker runs out.
• We use wet protein transfer (500 mA for 1 h), in a transfer buffer without SDS or alcohols, and nitrocellulose membranes with 0.45 μm pores. Other transfer conditions may be suitable but have to be optimised by the experimenter.
• Upon transfer, membranes should be stained with Ponceau S and image-scanned for future reference. To block membranes, incubate in PBS + 0.05% Tween (PBST) with 5% skimmed milk powder for 1 h at room temperature.
• *Note:* 4H8 is the most commonly used primary antibody for detection of ubiquitylated RPB1, as it recognises most forms of RPB1 (but preferentially the phosphorylated form) with high affinity (4H8 is available from a number of commercial providers). Other RBP1 antibodies can be used as well, but they will generally give lower signal intensity. In addition to RPB1, a housekeeping protein such as human Vinculin (∼120 kDa), or yeast Tub1 (50 kDa) or Pgk1 (45 kDa) should be probed against as well to control for loading in the inputs.
• From this step, standard Western blot procedures should be applied. For 4H8, we use a 1:10,000 primary antibody dilution (stock concentration 1 mg/ml).

### 3.8 Considerations for Dsk2/MultiDsk pulldown assay

• *Timing – when to look for RNAPII ubiquitylation after a stimulus?* In the case of UV-irradiation, RPB1 poly-ubiquitylation can be detected as early as 10 min after irradiation. The poly-ubiquitylated smear peaks around 30 min after UV, and is present up to a few hours after UV-irradiation (Fig. 2B), in both mammalian and yeast cells. As RPB1 molecules are getting ubiquitylated, they will also start to be degraded (see next section). The decrease in poly-ubiquitylated smears that is observed at time-points after ∼60 min can be attributed to a decrease in transcription (only elongating RNAPIIs stopped by DNA lesions are ubiquitylated and degraded) [12] and to a reduction in overall RPB1 levels in the cell, rather than to a loss of ubiquitylation activity (Fig. 2B).
  The dynamics of RPB1 poly-ubiquitylation described above applies to wild type cells, but various perturbations such as gene knock-outs, knock-downs, or mutations may affect this behaviour. For example, depletion of the E3 ubiquitin ligase NEDD4 affects RPB1 poly-ubiquitylation in early stages of the UV-response (10–20 min) whereas at later timepoints after UV, little or no change is observed between NEDD4-depleted and wild type cells [12]. We therefore recommend that the pilot experiments always include multiple timepoints, spanning both early (10–30 min) and late timepoints (a few hours) after a perturbation.
• *Use of proteasome inhibitors.* If optimal timepoints are chosen (see previous point), the use of proteasome inhibitors such as MG132 is not required to observe human RPB1 poly-ubiquitylation. However, proteasome inhibitors can of course be used, if the experimental question requires so (for example to confirm that disappearance of the smear at later timepoints was indeed due to proteasomal degradation of the poly-ubiquitylated RPB1 species). In this case, we typically use a 1–3 h pre-incubation with 5 μM MG132. It is important not to add a very concentrated MG132 stock solution directly to cell culture media as precipitates will form – we typically dilute the original 50 mM MG132 stock (in DMSO) 1:500 (in warm media) and add the diluted stock to cells. In yeast, MG132 is used only in the protein extraction buffer.
• *Pulldown efficiency.* When performing a Dsk2/MultiDsk pulldown, especially for the first time, it is recommended to run the input, unbound and bound fractions on protein gels and probe with an anti-ubiquitin antibody. This will show whether similar amount of input material was used, and reveal how efficient the pulldown is under the given conditions. The amount of beads and lysate can be varied to find optimal pulldown conditions (e.g. see [38]).
• *Replicates*. Even though Dsk2/MultiDsk pulldown is highly efficient, it suffers from some technical variability as any other method. We therefore recommend running each experiment as three independent, biological replicates. This becomes particularly important if one expects only moderate differences in RPB1 poly-ubiquitylation between different samples.
• *Alternative uses*. Dsk2/MultiDsk pulldown can be coupled with applications other than Western blot, such as mass spectrometry to identify ubiquitylated proteins proteome-wide, or further purifications (see below). Upon pulldown, bound proteins together with Dsk2/MultiDsk itself can be eluted with glutathione [38]. Alternatively, Dsk2/MultiDsk can be chemically crosslinked to the sepharose beads prior to pulldown, and upon pulldown, the bound proteins (without Dsk2/MultiDsk) can be eluted, for example by boiling in Laemmli buffer.

### 3.9 Troubleshooting Dsk2/MultiDsk pulldown assay

• *No signal in the pulldown fractions or variable signal between pulldown samples where it is not expected due to biology.* Possible reasons:
  o Low pulldown efficiency – was the efficiency checked with anti-ubiquitin antibody?
  o Antibody not working in Western blot – is the expected band(s) detected in the input samples?
  o Deubiquitylation of proteins in the extract – has freshly-made NEM been added to lysis buffer and pulldown reactions?

## 4 Tracking RNAPII degradation

*Background:* RPB1 degradation can be assessed by Western blot analysis, with some specific considerations outlined below.

### 4.1 Method: analysing RNAP II degradation by Western blot

Either whole cell extracts or chromatin fractions can be used to assess RPB1 degradation in mammalian cells. Most extraction protocols will be suitable as long as protease inhibitors, phosphatase inhibitors and freshly made NEM are added.

For yeast cells, the protocol for preparing lysates for Dsk2-pulldown can be used. Alternatively, it may be preferable to use a quicker lysis method that requires significantly less cells, such as alkaline quick whole cell extract preparation [40]. Briefly, 1–2 × 10^7^ cells are pelleted and resuspended in 100 μl of 100 mM NaOH for 5 min at room temperature. Cells are pelleted and the pellet resuspended directly in 50 μl of 1.5X Laemmli loading buffer and boiled for 5 min, before placing on ice. Samples are re-heated and spun at 14,000 g for 1 min before loading on SDS-PAGE gels (for measuring Rpb1 degradation, we usually load 8 μl per sample). Using this method, it is not possible to measure the protein concentration and so it is important to ensure that an equal number of cells is processed per condition, and to employ a non-degraded control protein.

**Fig. 3:**
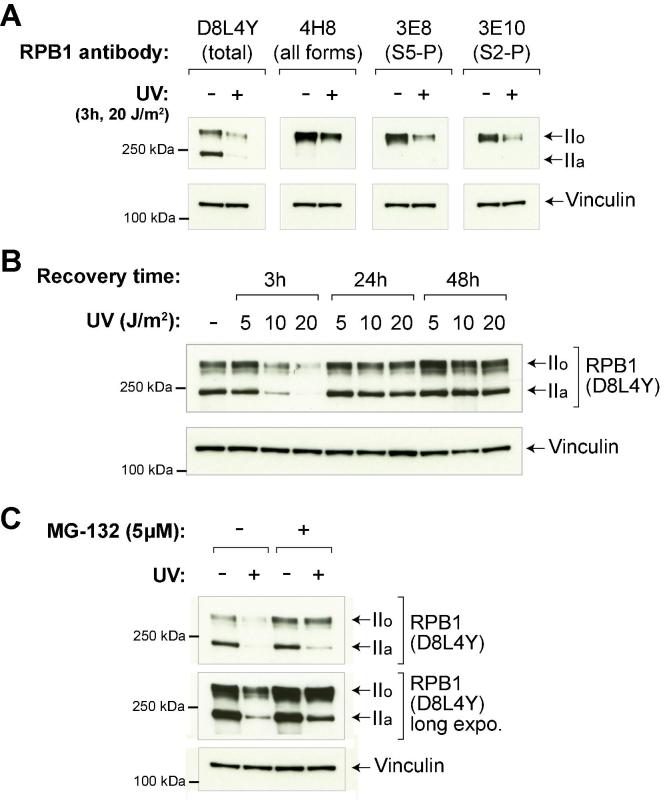
Analysis of RNAPII degradation. (A) Comparison of different RPB1 antibodies for detection of UV-induced RPB1 degradation. Identical samples (lysates from HEK293 TRex flpIn cells, either untreated or irradiated with 20 J/m^2^ UVC and collected 3 h post-irradiation), were run in quadruplicate on the same gel, and the membrane was cut and probed with different antibodies, recognising either total RPB1 (D8L4Y), all forms of RPB1 (with preference for the phosphorylated form) (4H8), Ser 5-phosphorylated RPB1 (3E8), or Ser 2-phosphorylated RPB1 (3E10). (B) Dose-dependent RPB1 degradation upon UV irradiation. HEK293 TRex flpIn cells were irradiated with 5, 10 or 20 J/m^2^ UVC, and samples were taken at different timepoints after irradiation. (C) Use of the proteasome inhibitor MG132 to confirm proteasomal degradation of RPB1. HEK293 TRex flpIn cells were pre-treated with 5 μM MG132 for 3 h, UV irradiated with 20 J/m^2^ UVC, and samples were collected 3 h after irradiation.
• Important note: Antibodies raised against the N-terminal of human RPB1 will *not* recognise yeast Rpb1. Unlike human RPB1, yeast Rpb1 migrates as a single band on polyacrylamide gels. To assess yeast Rpb1 degradation, 4H8 antibody can be used. Alternatively, yeast strains with tagged, endogenous Rpb1 can be generated, with anti-tag antibodies used to analyse Rpb1 degradation. We have successfully used this technique with a standard 3 × HA C-terminal tag on Rpb1 which was generated by homologous recombination [41].

To perform the Western blot:

• 10–50 μg of total protein should be loaded. It is important that equal amounts of protein are added to compare the different samples.
• To resolve unphosphorylated and phosphorylated RPB1, 3–8% Tris-Acetate gels should be used, and electrophoresis continued until the 75–100 kDa marker position runs out. Alternatively, 4–15% TGX gels can also be used as long as the 75–100 kDa marker protein runs out of the gel.
• We use wet protein transfer (500 mA for 1 h), transfer buffer without SDS or alcohols, and nitrocellulose membranes with 0.45 μm pores. Other transfer conditions may be suitable but have to be tested by the experimenter.
• Upon transfer, membranes should be stained with Ponceau S and scanned for future reference. To block membranes, incubate in PBS + 0.05% Tween (PBST) with 5% skimmed milk powder for 1 h at room temperature.
• From this step, standard Western blot procedures should be applied.
• The choice of antibody for detecting RPB1 degradation is very important. We recommend using phospho-unbiased antibodies, such as those raised against RPB1 N-terminus (N-20, D8L4Y, ARNA-3), when analysing RPB1 degradation. This is because stimuli such as UV-irradiation can cause RPB1 phosphorylation changes as well as degradation. Thus, interpreting changes of abundance of only the phosphorylated forms of RPB1 might be difficult. (Fig. 3A, also see “Considerations” below).

### 4.2 Considerations

• *Timing – when to look for RNAP II degradation after a stimulus?* The extent of RPB1 degradation will depend on the intensity of the stimulus – in the case of UV, the dose of irradiation. Doses between 10 and 20 J/m^2^ will induce almost complete degradation of RPB1 in HEK293 cells around 3–6 h after UVC-irradiation. Lower doses of UV will have similar dynamics but will cause degradation of a smaller percentage of RPB1 molecules (Fig. 3B). The amount of RPB1 will typically recover to original levels around 24–48 h after UV-irradiation (Fig. 3B). To observe UV-induced Rpb1 degradation in yeast, hourly time points are typically taken for 4 h. In contrast, when using 4-NQO in yeast, the time course is typically every 30 min for 120 min total (this time-course is appropriate for 10 μg/ml 4-NQO; for other concentrations the time course will need to be optimized).
• *Coinciding RPB1 phosphorylation changes –* Upon UV-irradiation of human cells, a characteristic RPB1 “hyperphosphorylation” can be observed, involving an almost complete shift of the unphosphorylated IIa band to the hyper-phosphorylated state (IIo). Importantly, two opposing forces will affect the total amount of phosphorylated RPB1 (IIo form) observed: continued CTD phosphorylation will increase its levels, while degradation will decrease it. Using phosphorylation-independent antibodies, such as N-20, D8L4Y or ARNA-3, raised against RPB1 N-terminus, will therefore provide an objective image of *total* RPB1 levels, influenced by degradation (Fig. 3A). As noted before, to assess Rpb1 degradation in yeast, either tagged-Rpb1 or 4H8 antibody can be used.
• *Using proteasome inhibitors –* Overall levels of a protein are determined by its synthesis rate and its destruction rate. In order to verify that the depletion of RPB1 is truly caused by proteasomal degradation, MG132 or another proteasome inhibitor can be used (Fig. 3C). In human cells, we typically use 5–10 μM MG132, added a few hours before UV treatment. In yeast, MG132 is used only in the lysis buffer. Wild type yeast are not sensitive to MG132 as it cannot efficiently enter the cells [42]. Mutant strains and chemical strategies have been described that increase the uptake of MG132 [43], [44] leading to accumulation of high molecular weight ubiquitin conjugates upon MG132 treatment [26]. However, these methods are not suitable for analysis of Rpb1 ubiquitylation and degradation because MG132 blocks proteasome-mediated Def1 processing, a critical pathway that is required for Rpb1 poly-ubiquitylation and degradation [26].
• *Using protein synthesis inhibitors –* In human cells, UV-induced RPB1 degradation can be observed even without using the protein synthesis inhibitor cycloheximide, showing that degradation of RPB1 is more rapid than synthesis of new RPB1 molecules. Indeed, without cycloheximide, human cells recover RPB1 levels only 24–48 h after a medium to strong dose of UV-irradiation. In contrast, yeast cells are capable of recovering Rpb1 levels much faster than human cells – a few hours after UV. Depending on the strain background, this can often make it difficult to capture the correct timepoint(s) to detect Rpb1 degradation. Addition of cycloheximide (25 μg/ml) to yeast cells immediately before UV treatment largely alleviates this problem. We have successfully observed Rpb1 degradation both in the absence [9] and presence [26] of cycloheximide and it is important to be aware that the profiles will look different depending on which method is chosen.
• *Replicates –* As with any experiment, we recommend performing the analysis in at least three independent, biological replicates. If expected/observed differences between the samples are moderate, image analysis (using programs such as ImageJ) can be used to quantify these differences. Loading controls such as Vinculin (human cells), and Tub1 or Pgk1 (yeast) can be used to normalise the data.

### 4.3 Troubleshooting RPB1 degradation analysis

• *No RPB1 degradation in samples where it is expected*. Possible reasons:
  o Unequal loading. Ensure that equal amounts are loaded based on the control protein.
  o Insufficient dose of the stimulus – try increasing the dose of the stimulus (see Table 1) and test different timepoints, and – if using UV-irradiation- check that the UV-irradiator and UV meter are working correctly.
  o Old reagents – some agents, such as cisplatin, need to be made fresh every time, otherwise they are hydrolysed or lose activity in solution.
  o For both of the above, it is useful to also blot for markers of the given stimulus (e.g. DNA damage markers such as γH2AX for cisplatin or UV) to check that the treatment has worked.
• *Additional bands or background in Western blot apart from IIo and IIa*. Possible reasons:
  o Non-specific degradation of proteins – have protease inhibitors been added to the lysis buffer? Has the sample been kept cold at all times? Have the samples been boiled in Laemmli buffer for too long (more than 7–8 min)?
  o Primary/secondary antibody background – try using different antibodies.
• *Phosphorylated RPB1 band (IIo) appears too weak*. Possible reasons:
  o Non-specific dephosphorylation of proteins – has phosphatase inhibitor been added to the lysis buffer?

## 5 Outlook – investigating specific ubiquitylation sites on RNAPII

### 5.1 Mapping individual ubiquitylation sites on RNAPII

Apart from targeting proteins for degradation, ubiquitylation can also act as a signalling module, in the form of mono-ubiquitylation or ubiquitin chains with linkage other than K48 [45]. It is thus possible that RNAPII is marked with several, different kinds of ubiquitylation. The functional importance of different ubiquitylation sites on RNAPII is typically not known.

While it is possible to couple Dsk2/MultiDsk pulldown with mass spectrometry, if Dsk2/MultiDsk are first crosslinked to the beads (see Section 3.8), this approach will typically only identify ubiquitylated proteins, but not necessarily the exact sites of ubiquitylation on each target protein. To map individual sites of ubiquitylation, enrichment of peptides with a Ubiquitin Branch Motif (K-ε-GG) antibody can be used. This principle is often referred to as diGly immunoprecipitation (diGly IP) [46], and can be coupled to stable isotope labelling with amino acids in cell culture (SILAC) labelling to enable quantitative profiling of ubiquitylation sites proteome-wide (see, for example, [33], [47], [48]). It is important to note that the diGly antibody enriches for three types of post-translational modifications indiscriminately: ubiquitylation, neddylation and ISGylation. However, given the abundance of the three in the cell it is fair to assume that most diGly peptides stem from a ubiquitylation event.

Building on the above, we have established a few modifications to this procedure in human cells. Briefly, we employed a SILAC-based quantitative proteomics approach. Cells are grown in media containing heavy- or light-isotope-containing amino acids to achieve light/heavy labelling of proteins [49].

Cells are then pelleted and lysed by addition of 9 cell-pellet volumes of 9 M urea solution (9 M urea in 20 mM Hepes pH 8.0) and sonicated to facilitate extraction of chromatin-bound proteins. Optionally, 100 U/ml of benzonase may be added to the lysate prior to sonication to facilitate the process. Lysates are then cleared by centrifugation, and equal amounts of protein from each sample (typically 30 mg) is subjected to LysC and tryptic digests, as specified by the Ubiquitin Remnant Motif (K-ε-GG) Kit manufacturer. After C18-reverse phase purification using Sep Pak cartridges, ubiquitylated peptides are enriched using K-ε-GG antibody conjugated to protein A agarose beads. 40 μl of antibody-coupled beads are used per 30 mg of input sample. Enriched peptides are eluted with 0.15% TFA, lyophilized, and subjected to SCX (strong cationic exchange) or basic reverse phase fractionation [33], [48], [50]. The fractions are then analysed using LC MS/MS in combination of nanoLC and a suitable mass spectrometer (e.g. LTQ-Orbitrap Velos Q-Exactive Orbitrap or an Orbitrap Fusion Lumos) [48], [50], [51]. This approach should typically yield between 5000 and 10,000 ubiquitylation sites per experiment. Using this method, multiple UV-induced ubiquitylation sites can be identified on RNAPII [33], [47], [48]. Additionally, for analysis of a larger number of different conditions, the diGly enrichment principle can be coupled with multiplexing procedures such as Tandem Mass Tag (TMT) Systems [51] instead of SILAC labelling.

For the diGly IP protocol in yeast cells please refer to a study by Espenshade and colleagues [52]. In this study diGly IP has been performed on previously enriched ubiquitylated proteins, isolated from yeast cells expressing His-tagged ubiquitin using nickel affinity purification. Therefore, it might be possible to apply the same principle in yeast or human cells using Dsk2/MultiDsk enrichment of ubiquitylated proteins and subsequent diGly IP, which might result in an even better detection of ubiquitylation sites than using diGly IP alone.

We envisage that mapping individual sites of ubiquitylation on RNAPII using methods outlined above will be crucial for understanding the molecular mechanisms that mediate RNAPII degradation under different conditions.

### 5.2 Model systems to study RNAPII PTMs, including ubiquitylation

The RNAPII complex harbours numerous post-translational modifications (PTMs), the most famous being CTD phosphorylation. These modifications are a platform for regulating RNAPII behaviour at different stages of transcription. Even for the most well studied RNAPII PTMs, such as phosphorylation of Serine 5 or Serine 2 within the CTD hepta-peptad repeats, novel roles are constantly emerging (see, for example, [53]). This only highlights how much is still unknown, especially regarding other, more difficult-to-study RNAPII modifications such as ubiquitylation.

When studying PTMs, the first approach is often to mutate the modification acceptor site on the target protein. To prevent ubiquitylation of a specific lysine residue, lysine (K) is mutated to either the conservative arginine (R) or, less optimally, to alanine (A). While K → R/K → A mutant proteins can be tagged, overexpressed and purified for *in vitro* assays, the challenge remains of how to study the functions of a particular mammalian PTM *in vivo*. To elucidate the roles of a PTM *in vivo*, the endogenous wild type protein has to be replaced with the mutated version. For human RNAPII, the most commonly used model system to achieve this is the α-amanitin resistance system [54], [55]. α-amanitin is a mushroom amatoxin that binds the catalytic site of RNAPII. This blocks its forward translocation [56] and induces rapid RPB1 degradation [57]. A specific mutation in RPB1 has been identified (N793D) that confers resistance to α-amanitin [58]. This mutation has been exploited to generate α-amanitin-resistant RPB1 constructs that also harbour CTD deletions, or desired mutations in PTM sites, for example [54], [55], [59]. By transiently transfecting these constructs into cells, and treating with α-amanitin, the “switchover” from endogenous (‘α-amanitin-degraded’) to the mutated, α-amanitin-resistant RNAPII is achieved. This and other systems like it can be used to investigate the *short-term* effect of often lethal mutations in the *RPB1* (*POLR2A*) gene. For studying long-term effects of a given RNAPII PTM, alternative “switchover” systems may be developed by targeting the endogenous RNAPII loci with siRNA/shRNA or CRISPR-mediated knock-outs, while at the same time expressing PTM-mutant forms from stably integrated sites in the genome. Finally, it may be possible to target the endogenous RNAPII loci with CRISPR-mediated knock-in point mutations in the desired PTM sites when these mutations are not lethal.

## 6 Conclusions

Affinity resins based on ubiquitin binding domains such as Dsk2 or MultiDsk provide a robust and easy method to enrich endogenous ubiquitylated proteins in their native state. Importantly, these strategies are suitable not only for analysis of RNAPII ubiquitylation, but also of any other ubiquitylated protein, and can be coupled either to Western blot or other analyses, such as further purification or mass spectrometry. While Dsk2 protein alone has some preference towards K48-linked ubiquitin chains, MultiDsk binds different kinds of ubiquitin chains equally well. Nonetheless, both resins successfully enrich poly-ubiquitylated RNAPII species, although Dsk2 resin is preferable for this purpose as it does not enrich the mono-ubiquitylated form to an extent where it affects the visualization of the often weaker RPB1 poly-ubiquitylation signal.

RNAPII ubiquitylation triggered by transcription stress, such as that caused by bulky DNA lesions, targets RNAPII for degradation by the proteasome. The kinetics and peak time of RNAPII degradation will depend on the type and intensity of the stress stimulus (DNA damage, NTP depletion, RNAPII inhibitors). Some of these agents also induce changes in RNAPII phosphorylation patterns, thus using the phosphorylation-unbiased antibodies for detection of total RNAPII levels is recommended.

At present, it is unclear which exact sites on RPB1 are modified by ubiquitylation, and by what type of ubiquitin chains, and how such modification affects RNAPII function. As multiple E3 ubiquitin ligase pathways have been implicated in targeting RNAPII, this question becomes increasingly important. Advanced strategies for profiling individual ubiquitylation sites in the proteome and generating suitable model systems to study those, will be crucial to understand how the “ubiquitin code” regulates the behaviour and fate of RNAPII.

## Figures and Tables

**Table:** 

UV source	Stratalinker or similar	Stratagene
UV radiometer VLX-3W	513000111	Progen Scientific
UV Sensor SX-254 Radiometer Sensor 254 nm	549000011	Progen Scientific
Bulbs Stratalink 2400 Bulbs, 365 nm 254 nm	400079	Agilent
α-amanitin	sc-202440A	Santa Cruz
6-azauracil (6-AU)	A1757	Sigma
Cisplatin	P4394	Sigma
*N*-Ethylmaleimide (NEM)	04260	Sigma
MG132	10012628	Cambridge Bioscience
4-Nitroquinoline N-oxide (4-NQO)	N8141	Sigma
Glutathione Sepharose Beads	GE17-0756-01	Sigma
cOmplete(TM), EDTA-free Protease Inhibitor Cocktail	5056489001	Sigma

**Table:** 

RPB1 (4H8)	ab5408	Abcam (or similar)
Rpb1 NTD (D8L4Y)	14958S	NEB
RPB1 P-Ser2 (3E10)	04-1571	Merck Millipore
RPB1 P-Ser5 (3E8)	04-1572-I	Merck Millipore
α-Pgk1	459250	Invitrogen
Tubulin (TAT-1)	00020911	Sigma
Vinculin	V9131	Sigma
K-ε-GG (PTMScan kit)	5562	Cell Signaling Technology
